# 2′-O-Methylation of Ribosomal RNA: Towards an Epitranscriptomic Control of Translation?

**DOI:** 10.3390/biom8040106

**Published:** 2018-10-03

**Authors:** Piero Lo Monaco, Virginie Marcel, Jean-Jacques Diaz, Frédéric Catez

**Affiliations:** Univ Lyon, Université Claude Bernard Lyon 1, INSERM U1052, CNRS UMR5286, Cancer Research Center of Lyon, 69008 Lyon, France; piero.lomonaco@lyon.unicancer.fr (P.L.M.); virginie.marcel@lyon.unicancer.fr (V.M.)

**Keywords:** 2′-O-methylation, ribosomal RNA, fibrillarin, snoRNP, ribosome heterogeneity, mRNA translation

## Abstract

Ribosomal RNA (rRNA) undergoes post-transcriptional modification of over 200 nucleotides, predominantly 2′-O-methylation (2′-O-Me). 2′-O-Methylation protects RNA from hydrolysis and modifies RNA strand flexibility but does not contribute to Watson-Crick base pairing. The contribution of 2′-O-Me to the translational capacity of ribosomes has been established. Yet, how 2′-O-Me participates in ribosome biogenesis and ribosome functioning remains unclear. The development of 2′-O-Me quantitative mapping methods has contributed to the demonstration that these modifications are not constitutive but rather provide heterogeneity to the ribosomal population. Moreover, recent advances in ribosome structure analysis and in vitro translation assays have proven, for the first time, that 2′-O-Me contributes to regulating protein synthesis. This review highlights the recent data exploring the impact of 2′-O-Me on ribosome structure and function, and the emerging idea that the rRNA epitranscriptome is involved in translational control.

## 1. Introduction

Chemical modification is a major mechanism that regulates the properties and functions of biological molecules. RNAs are no exception and modifications are found in all classes of RNA and in all living organisms. To date, at least 171 types of modifications have been identified (Modomics database, http://modomics.genesilico.pl) [1]. Ribosomal RNA (rRNA) bases undergo several types of modifications either through the addition of chemical groups, such as methyl- or acetyl-groups, or through the isomerization of uridine into pseudouridine (ψ). The ribose is also subjected to modifications by the addition of a methyl group at its 2′ position (2′-O-methylation, 2′-O-Me). Moreover, several nucleotides are subjected to multiple sequential modifications. The number of modified nucleotides increases from 33 in bacteria to 112 in yeast and over 210 in humans, a tendency that parallels not only the growing size and complexity of the ribosome but also the increased complexity of translational programs [2,3,4]. Deciphering the molecular roles and biological functions of these modifications is one of the greatest challenges of modern biology. This review focuses on recent progress regarding rRNA 2′-O-methylation and its contribution to the functioning of human ribosomes and messenger RNA (mRNA) translation.

## 2. Adding 2′-O-Methylation to Eukaryotic rRNA: How, Where, and When?

**Figure 1:**
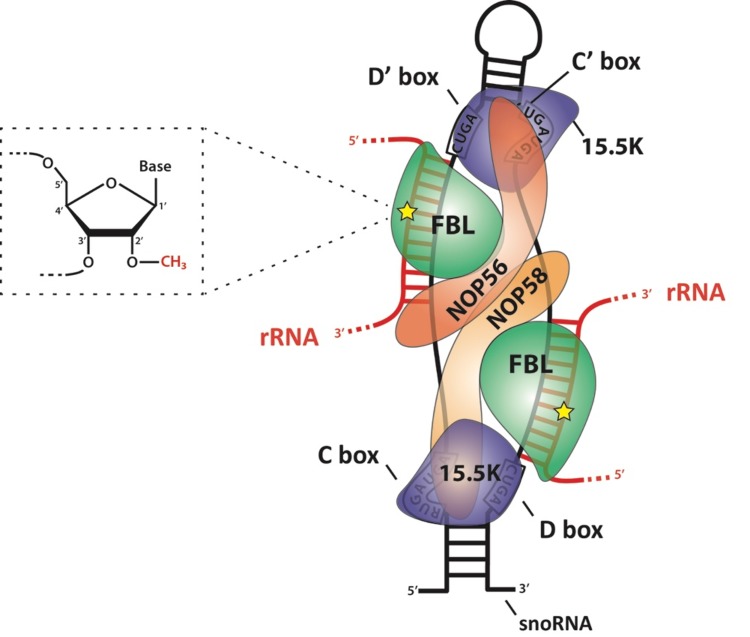
Schematic representation of the C/D box snoRNP complex, depicting the main features of the complex according to both biochemical data and structural studies using an archaeal complex. The C and D boxes are depicted with their consensus sequence. The substrate ribosomal RNA (rRNA, red) forms an RNA:RNA duplex with the snoRNA along 10–21 nucleotides (nt). The catalytic site of fibrillarin (FBL) faces the fifth nucleotide downstream of the D or D′ boxes (yellow star). The 15.5K protein specifically binds a k-turn motif. The NOP56:NOP58 heterodimer (which is a homodimer in archaea) forms two long alpha helices that are placed across the snoRNA:rRNA complex and contribute to locking FBL in its active position. rRNA: ribosomal RNA; snoRNA: small nucleolar ribonucleoprotein.

2′-O-Methylation is added to rRNA post-transcriptionally by ribonucleoprotein (snoRNP) complexes containing C/D box family small nucleolar RNAs (snoRNAs), thus termed C/D snoRNP complexes, in which the snoRNA defines the nucleotide to be methylated by sequence complementarity with rRNA (Figure 1). snoRNA-mediated guidance is also used to specify ψ sites (see [5]). This differs from stand-alone enzymes that directly ensure substrate specificity and represents the main mechanism of base modification. However, there are some exceptions, for instance, the acetylation of two bases of the yeast 18S rRNA, the modification of which is carried out by *Kre33*, and substrate specificity guided by snoRNAs snR4 and snR45 [6]. The assembly and composition of C/D snoRNP complexes have been reviewed extensively and is simply summarized herein [2,7,8,9]. In addition to one C/D box snoRNA, C/D snoRNPs contain the methyltransferase fibrillarin (FBL, Nop1 in yeast), the RNA binding protein 15.5K (also named NHP2L1, and Snu13 in yeast), and the heterodimer of two closely related proteins NOP56 and NOP58. Methyltransferase fibrillarin uses S-adenosyl methionine (SAM) as the methyl donor. C/D box snoRNAs are defined by two conserved sequences named C (RUGAUGA, where R is a purine) and D boxes (CUGA), which are usually present in duplicate (the second set is marked C′ and D′) and flank an antisense sequence to the targeted rRNA. C/D box snoRNPs are structured in a manner that positions the catalytic site of FBL directly opposite the nucleotide to be methylated, within the duplex formed by the guiding snoRNA and the targeted rRNA, precisely five nucleotides upstream of a D box (or D′) [10,11,12]. The archaeal C/D sRNP complex is the main model used to study this structure, due in part to the difficulty in assembling the eukaryotic snoRNP complex in vitro. Some controversy remains as to how the complex is specifically organized. Several studies have revealed how rRNA/snoRNA base pairing, 15.5K (named L7ae in archaea), and the NOP56/NOP58 dimer (Nop5 homodimer in archaea) are instrumental in positioning the FBL catalytic site and the methyl donor SAM within close proximity of the 2′-OH group to be methylated [12,13,14,15].

In humans, C/D box snoRNAs (or SNORD) are 70–120 nucleotides (nt) long and are mainly encoded within introns of coding genes, and processed from lariat introns upon mRNA splicing. Since many snoRNAs are encoded by genes associated with ribosomal biology, such as ribosome biogenesis factors or ribosomal proteins, it is expected that this genomic organization promotes co-regulatory mechanisms, although this remains to be demonstrated. C/D box snoRNP particles are assembled via a multi-step process, involving chaperone and helicase activities carried out by complexes such as R2TP [16,17,18,19], which ensures the loading of 15.5K, NOP58, NOP56, and FBL proteins onto the C/D box snoRNA. During this process, 15.5K will specifically bind to a k-turn structure within the C/D box. C/D box snoRNP assembly takes place in part within the Cajal bodies (one of the preeminent nuclear domains involved in small non-coding RNA maturation) before the complex migrates to the nucleolus where it operates at an early stage of ribosome biogenesis, likely within or at the edge of the dense fibrillar component.

Evaluating the timing of 2′-O-Me has been a long-standing issue. Early studies demonstrated that 2′-O-Me is a nuclear event [20]. More recently, in an elegant series of isotope labeling experiments, data fitting and mathematical modeling, Tollervey’s laboratory provided the first dataset showing that a large portion of 2′-O-Me occurs co-transcriptionally in yeast, with the 18S rRNA precursor being almost fully modified before its release from the RNA polymerase I (Pol I) complex [21]. Quantitative site-specific analysis comparing chromatin-associated RNA with mature rRNA further confirmed the general principle established by labeling assays [22]. In addition, it revealed that in yeast, even at a very early stage, before the 18S rRNA is cleaved from the pre-rRNA, all but one position of the 18S rRNA (18S-Am100) displayed over 80% of their final level of modification. Accordingly, the 25S rRNA sites are less methylated in the RNA Pol I-associated pre-rRNA [22]. Thus, the current knowledge on the timing of 2′-O-Me in yeast cells supports a general model in which rRNA is modified at a very early stage of ribosome biogenesis and is likely to be completed before rRNA is fully assembled as ribosomal pre-subunits. It remains to be determined whether such a model applies to human rRNAs, which display partially methylated nucleotides (see below).

In most cases, a 2′-O-Me position is modified by a unique C/D box snoRNA so that single SNORD/2′-O-Me associations can be defined. However, there are a few cases of combinatorial associations, such as C/D snoRNAs modifying several sites (e.g., SNORD50A modifies 28S-Cm2848 and 28S-Gm2863 in humans) or 2′-O-Me positions modified by more than one C/D snoRNA (e.g., 18S-Am668 by SNORD36A and SNORD36B, both encoded by the *RPL7A* gene in humans). How such SNORD combinations contribute to regulating 2′-O-Me remains obscure.

## 3. 2′-O-Methylation as a Source of Heterogeneity: All Ribosomes Are Not Created Equal

In line with other RNA chemical modifications, detecting and measuring 2′-O-Me remains challenging [23]. 2′-O-Me detection methods have previously been extensively reviewed and are only summarized herein [23,24,25,26]. Detection of 2′-O-Me is based essentially on two properties of 2′-O- methylated RNA. First, 2′-O-Me replaces the 2′-OH group, thus rendering methylated RNA resistant to hydrolysis compared to unmethylated RNA. Second, 2′-O-Me tends to block retro-transcription of RNA into complementary DNA (cDNA) when the reaction is performed under non-optimal conditions (e.g., low deoxy-nucleoside triphosphate (dNTP) concentration). Both properties have been used for site-by-site analysis and were exploited only recently for global mapping in a single experiment using RNA-sequencing based methods [16,19]. All RNA-sequencing based approaches provided qualitative mapping to identify 2′-O-Me positions and revealed novel perspectives in ribosome biology but also in the field of epitranscriptomics. Of note, the method called Nm-seq appears to be one of the most sensitive methods and allows the detection of 2′-O-Me even from scarce amounts of RNA, including mRNA and non-coding RNA [27]. This is achieved by means of iterative cycles of oxidative-elimination-dephosphorylation reactions, which sequentially eliminate 2′-unmodified nucleotides from 3′ to 5′ and leave the 2′-O-methylated sites intact. In the final step, dephosphorylation is prevented so that only RNA fragments with a 2′-O-methylated 3′ end possess a 3′-OH at their 3′ end, and can be ligated for library preparation. Consequently, 2′-O-Me sites generate a positive signal and not a lack of signal, in contrast to the methods based on the inhibition of retro-transcription (RT) reaction or of hydrolysis. Among these novel mapping methods, RiboMeth-seq has emerged as the only method that consistently detects all known 2′-O-Me sites in yeast and human rRNAs [22,28,29,30]. Most importantly, RiboMeth-seq is the only available method for the quantitative evaluation of 2′-O-Me levels at each position, thus providing information on potential 2′-O-Me variations and providing 2′-O-Me profiles [22,29]. At present, two different protocols have been published. The first one was reported by Nielsen’s group and the second alternative protocol, which uses very low amounts of starting material, was reported by Motorin’s group [22,28,29,30,31].

The repertoire of 2′-O-Me was recently updated by Krogh and colleagues and contains a list of 106 validated sites, as well as two additional strong candidate sites that currently lack clear evidence by mass spectrometry [28,30]. These 106 2′-O-methylated nucleotides were considered validated using several approaches: (1) biochemically [24]; (2) RiboMeth-seq; (3) mass spectrometry (if new or uncertain); and (4) verifying that at least one guide C/D box snoRNA had been attributed to the corresponding nucleotide environment. This list is expected to grow as nucleotides that are modified either at a low level or only in specific biological contexts have not yet been validated by several independent techniques. At present, several novel candidate nucleotides have been published using different high confidence 2′-O-Me mapping techniques [27,32,33]. In addition, a set of 14 novel 2′-O-Me positions were exposed based on rRNA:snoRNA interactions and unveiled by cross-linking immunoprecipitation (CLIP) with antibodies against FBL, NOP58, and NOP56 [34]. It is critical that the RNA-modification databases are updated because new 2′-O-Me sites (and other modifications) are being discovered and validated.

2′-O-Me was considered to be a constitutive and universal modification, leading to the alteration of each site in every ribosome of the cell. Such a dogma stemmed from the methods used that were unable to detect small differences in the level of modification [24,35], and from the observation that in yeast, one of the historical 2′-O-Me models, rRNA display complete methylation at almost all sites in all ribosomes. Yet, evidence of fractional methylation of 5.8S-Um14 was provided in early studies based on nuclease fragmentation of 5.8S rRNA in mammalian tissue and HeLa cells [36,37] and more recently in yeast at the 18S-Am100 position using DNAzyme-based hydrolysis and LC-UV-MS/MS analysis [38]. This view of the rRNA 2′-O-Me landscape has largely evolved based in part on RiboMeth-seq data that showed that some specific positions in yeast rRNA are partially methylated [22,29]. This was further supported by large-scale MS-based analyses and mung bean nuclease assays followed by RP-HPLC [39,40].

**Figure 2:**
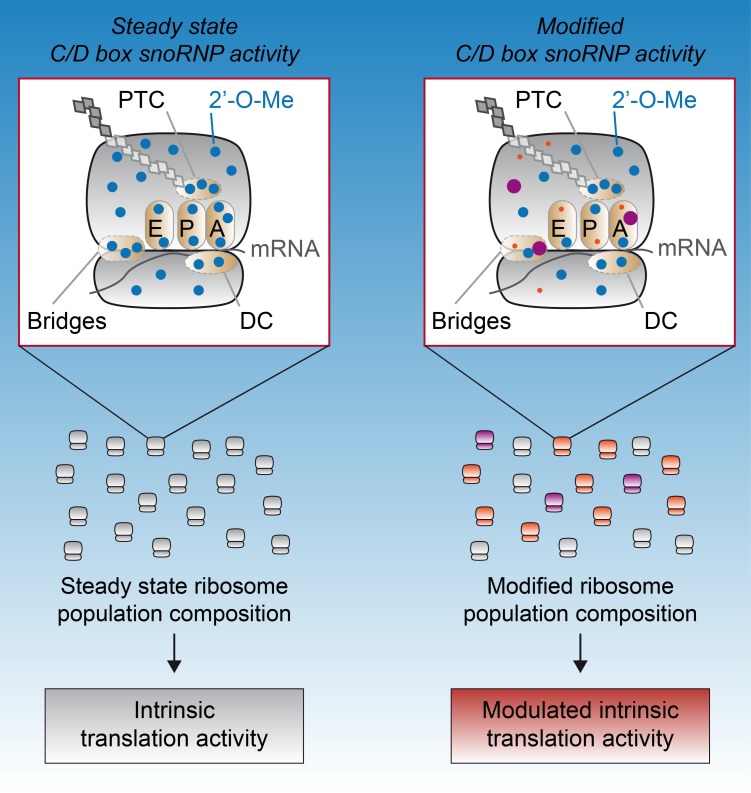
Model of 2′-O-methylation (2′-O-Me) profile modulation and its consequence on the intrinsic translational activity of ribosomes. In this model, we represent two possible states: a steady state and a modified state. Following a change in C/D box snoRNP activity (e.g., change in FBL or SNORD expression) during ribosome biogenesis, 2′-O-Me levels change in a site-specific manner as revealed in [30,31] (unchanged level: blue dots; decreased level: red dots; increased level: purple dots). 2′-O-Me is altered in key functional regions of the ribosome, including the inter-subunit bridges, the decoding center (DC), and the peptidyl-transferase center (PTC), the latter being located close to the transfer RNA (tRNA) binding sites (A: aminoacyl-tRNA binding site; P: peptidyl-tRNA binding site; and E: exit site) [25,26]. The ribosomal population changes in its composition and variety (gray, orange, and purple ribosomes). When tested in an in vitro cell-free translation assay, these ribosomal populations exhibit a differential translational activity [25]. mRNA: messenger RNA; snoRNA: small nucleolar ribonucleoprotein; SNORD: C/D box snoRNA gene.

Whether 2′-O-Me profiles of yeast rRNAs can vary under various physiological conditions remains to be explored. In humans, evidence of fractional 2′-O-Me was obtained using a semi-quantitative method based on the inhibition of the RT-reaction followed by qPCR, and revealed that 2′-O-Me levels at some sites varied among rRNAs from breast cancer cells at various stages of tumorigenesis and tumor progression [41,42]. Recent RiboMeth-seq based studies also demonstrated that methylation is partial (i.e., <80%), around a third of the 106 sites in exponentially growing cultured cells [28,30,31,34]. Having a number of partially methylated sites implies that the pool of ribosomes is heterogeneous in composition and that subpopulations of differentially methylated ribosomes co-exist (i.e., ribosomes that lack methylation at several sites). Hence, the complete set of 2′-O-Me is not required for rRNA assembly in a human ribosome as was initially assumed based on studies in yeast. This finding was corroborated by small interfering RNA (siRNA) results, which showed that FBL knockdown resulted in the production of ribosomes displaying up to 50% less 2′-O-Me [30]. Importantly, these data demonstrate that cells tolerate the production of partially methylated ribosomes (Figure 2). Overall, 2′-O-Me contributes to shaping a wide variety of ribosomes, thus creating ribosomal heterogeneity.

Mechanistically, the regulation of 2′-O-Me profiles is uncertain. siRNA knockdown studies unexpectedly revealed that changes in the FBL level result in a site-specific decrease in 2′-O-Me instead of a global and homogeneous decrease across all 2′-O-Me sites [30,31]. This indicated that the mechanisms regulating 2′-O-Me act in a site-by-site manner rather than globally. A likely regulatory mechanism resides in the modulation of the level of methylation at corresponding sites through changes in the expression of some C/D snoRNAs, depending on physiopathological conditions. Indeed, several examples have been reported in cancer, including significant changes in SNORD expression in leukemia and lung cancer [43,44,45,46]. Some of these were shown to contribute to tumorigenesis, such as SNORD50 in lymphoma and prostate cancer or SNORD78 in lung cancer [47]. However, their impact on modulating 2′-O-Me levels at the associated rRNA site remains to be addressed. Although these examples illustrate putative variations of 2′-O-Me via changes in snoRNA expression, the first analyses comparing 2′-O-Me profiles and snoRNA expression levels in cultured cells using omics approaches failed to establish any global correlation [28,31], thus suggesting that C/D box snoRNA levels may not be the primary 2′-O-Me regulatory mechanism. Additional mechanisms should be considered, such as the variable catalytic efficacy of individual C/D box snoRNPs. Post-translational modifications of FBL or its partners [48] could also contribute to this regulation, together with the structure of the snoRNA itself, since in vitro methylation assays revealed that D and D′ boxes can display variable catalytic efficacy and that the complementary sequence and its flanking sequences also influence the activity of the complex [12,15,49]. Another possibility is that additional factors might regulate the core snoRNP complex and its function. For instance, it was shown that uL13 protein (also named RPL13a) contributes to rRNA 2′-O-Me and can be pulled down with FBL and the snoRNA U15 [50]. Finally, it should be noted that the nucleolus is a very dense nuclear domain in which the probability of molecular interactions can be either reduced due to competitive binding to the same substrate or facilitated by a molecular crowding effect [51,52]. Whether this particular nucleolus environment influences the probability of snoRNPs finding their target and regulating 2′-OMe pattern remains to be explored.

The demonstration that 2′-O-Me is not constitutively added in all ribosomes and that cells tolerate the production of partially modified ribosomes represents major evidence of ribosome plasticity through composition variation. It is now critical to determine whether 2′-O-Me-mediated ribosomal heterogeneity takes place in normal and pathological tissues. Interestingly, genetically encoded variations in ribosome composition have been reported for ribosomal proteins, but they remain to be identified in rRNA [53,54].

## 4. 2′-O-Methylation Contribution to Ribosome Structure and Function

A key, yet unresolved issue is determining to what extent the presence or absence of 2′-O-Me modulates rRNA folding, dynamics, and interactions with RNAs (including rRNA, mRNA, tRNA) and ribosomal proteins. 2′-O-Me is one of the RNA modifications that does not directly alter the Watson-Crick laws governing base pairing, making its contribution to RNA structure and function even more complex to anticipate. In addition to protecting RNA from hydrolytic cleavage, a well-known consequence of 2′-O-Me is the stabilization of the nucleotide conformation (i.e., the C3′-endo puckering of the ribose moiety), which is favored in RNA strands due to the OH group [55]. In addition, 2′-O-Me restricts the rotational freedom of 3′-phosphate, thus potentially restricting RNA strand conformation and flexibility. Similar consequences apply to the RNA duplex. Indeed, the 2′-O-methylated RNA duplex displays hydration patterns, helix winding, and base tilting that are slightly different from non-2′-O-methylated RNA duplexes and indicate that 2′-O-Me may influence RNA helix flexibility [56,57]. 

There are no published follow-up studies focusing on the role of 2′-O-Me on RNA conformation, such as single strand rRNA or rRNA:protein complexes, that are found within ribosomes, and the field has thus not been updated for several years. Fortunately, recent progress in structural analysis of entire ribosomes has enabled the visualization of some rRNA modifications and unraveled their involvement in molecular interactions. Steitz’s laboratory reported a crystal structure of bacterial ribosome at a 2.3–2.5 Å resolution revealing several rRNA modifications including 2′-O-Me, and showed that they establish contact with ribosome ligands between the ribosomal subunits and within rRNAs [58]. For example, 2′-O-Me contributes to hydrophobic interactions between nucleotides, and 2′-O-methylated nucleotides, such as 23S-Gm2251 and 23S-Um2552, establish contacts with the CCA-end of tRNAs located both in A and P sites. Notably, these modifications are highly conserved across species, suggesting that they play an important role in shaping ribosome structure. There has also been a dramatic improvement in the resolution of the structure of large complexes using cryo-electron microscopy (cryoEM), which has granted access to the structure of human and other eukaryotic ribosomes [59,60,61]. Using improved cryoEM and refinement methods during image processing, Klaholz’s laboratory obtained structures at a resolution of up to 2.5 Å for human ribosomes and visualized 130 rRNA modifications including 57 2′-O-Me [61]. This study further demonstrated the role of 2′-O-Me in local and global stabilization of the rRNA structure, particularly in more flexible rRNA regions, such as bulges, hairpin loops, and rRNA-specific structural elements, such as k-turn or A-minor motifs. Overall, these studies have supported a contribution of 2′-O-Me and other rRNA modifications to the stabilization rRNA folds and the establishment of interactions not provided by non-modified nucleotides within functionally important regions of the ribosome. However, determining the structural impact of 2′-O-Me by comparing ribosomes carrying either methylated or non-methylated nucleotides will be essential. In yeast, footprinting of rRNA previously demonstrated that removal of 2′-O-Me and pseudouridylation alters the accessibility of rRNA [62]. Thus, data on 2′-O-Me localization within the ribosome and on their contribution to rRNA structure strongly support that 2′-O-Me contributes to ribosome function.

The role of 2′-O-Me on ribosome functioning and mRNA translation was initially explored in yeast in a series of studies in which both 2′-O-Me and ψ were selectively removed from key regions of the ribosome, such as the subunit interface, the peptidyl-transferase center, and the decoding center by introducing mutations into the corresponding snoRNA guide [62,63,64,65]. Similar observations were made for 2′-O-Me and ψ individually, and led to the conclusion that the lack of chemical modifications in at least two to three positions was necessary to observe an impact on cell growth and on the protein synthesis capacity of yeast cells. This observation led to the belief that individual 2′-O-Me sites were not essential to ribosome functioning. Nevertheless, additional data in yeast has indicated that 2′-O-Me contributes to regulating translational activity of the ribosomes, and may selectively affect translation rather than regulating bulk protein synthesis. Indeed, it was shown that 2′-O-Me impacted translation reliability and reproducibility and appeared to modify yeast phenotype when cells were exposed to antibiotics, stresses, or poor growth conditions [66,67]. These data suggested that 2′-O-Me might regulate translation of a subset of mRNAs, a hypothesis that is now supported by studies in metazoan models.

While single SNORD knockout had no or little impact on yeast cells, knocking down the expression of single snoRNAs had severe consequences on zebrafish embryonic development, suggesting that complex metazoan genetic programs require the full potential of ribosome 2′-O-Me [68]. Yet, this conclusion may have been hasty since lack of 2′-O-Me was verified at only one site (U26 target) and further experiments were not carried out to exclude snoRNA host gene deficiency. The role of 2′-O-Me in regulating translation of selected mRNAs has been progressively demonstrated, first by correlative observations between altered 2′-O-Me profiles and translation reporter assays in breast cancer models [41,42], and second in models in which expression of the rRNA methyltransferase FBL was knocked down [30,42,50,69]. Interestingly, modulation of FBL expression impacted mRNA translation initiated from internal ribosome entry sites (IRES). Internal ribosome entry site elements are highly structured RNA sequences that are able to recruit the small ribosomal subunit and initiate translation in a process that short-cuts the cap structure at the 5′ end of mRNAs. This mode of initiation is highly stimulated under stress when cap-dependent translation is reduced or inhibited, such as in response to heat shock, hypoxia, or viral infection. This concerns many cellular mRNAs that are key to cell behavior and contain IRES elements, including growth factors and receptors (e.g., *IGF1R*), apoptosis regulators (e.g., *XIAP*), oncogenes (e.g., *c-Myc*), and tumor suppressors (e.g., *TP53*) [70]. Using reporter assays, several independent studies established that modulation of FBL expression differentially impacted translation from various IRES elements, suggesting that 2′-O-Me might regulate mRNA selection by the ribosome. Of note, altering the pseudouridylation content of ribosomes also led to changes in IRES-dependent translation that differed from the one observed for 2′-O-Me, and further support that the two types of rRNA modifications modulate translation, although with distinct regulatory outcomes. In addition to IRES-containing mRNAs, a ribosome profiling analysis demonstrated that FBL modulation affects the translation of several other mRNAs, thus expanding the subset of mRNAs the translation of which can be regulated by 2′-O-Me [30]. Interestingly, mRNA translation was either increased or decreased in response to 2′-O-Me profile modulation, indicating that 2′-O-Me contributes to both stimulatory and inhibitory mechanisms [71]. These data suggest that modulation of FBL expression, and therefore of 2′-O-Me profile, likely impacts several regulatory mechanisms that remain to be identified [30].

Importantly, alterations in translational regulation observed in previously described cellular assays could be attributed to ribosomes. This was shown by means of an in vitro cell-free translation assay, in which purified ribosomes were the only variable parameter [30,72]. While ribosomes exhibiting an altered 2′-O-Me profile displayed similar translational activity on mRNA carrying a GAPDH or actin 5′-UTR compared to control ribosomes, they exhibited a decreased ability to initiate translation from the dicistrovirus IRES elements [30]. Thus, 2′-O-Me modulates the intrinsic ability of ribosomes to initiate translation in a 5′-UTR-dependent manner (Figure 2). Of note, a similar observation was made with ribosomes carrying an altered ψ content, further strengthening the central role of rRNA modifications in ribosome functioning and translational control [73]. Further exploration of in vitro cell-free assays [72,74] should help decipher which steps of the translation process are controlled by rRNA chemical modifications and identify target mRNAs. Altogether, these studies demonstrate that 2′-O-Me represents a novel molecular support for mRNA translation and contributes to the functional specialization of the ribosome. Deciphering how 2′-O-Me and other RNA modifications regulate translation will be a challenge in the coming years. Indeed, the precise molecular mechanisms governing the translational efficacy of a given mRNA remains partially understood. mRNAs are associated with many factors (cap-binding complex, RNA binding proteins, microRNAs) that modulate the recruitment of the 40S and 60S subunits. The ribosomal subunits play a significant regulatory role in this process, at least through subunit-bound factors. For example, the 40S-associated eIF3 "canonical" translation initiation complex was shown to carry a selective preference toward particular mRNA 5′-UTR sequences, thus modulating the translational efficacy of mRNA subsets [75]. Some 2′-O-Me rRNA may modulate the ribosome-bound proteomes that will either favor or restrict the recruitment of specific mRNA subsets. Another possibility is that 2′-O-Me regulates the direct interaction of the mRNA complexes with rRNA as suggested for mRNAs on which translation can be initiated in a cap-independent manner [70].

## 5. Perspective: Towards an rRNA-Based Epitranscriptomic Control of mRNA Translation

rRNA 2′-O-methylation was considered to be a constitutive chemical modification that optimized the functioning of higher eukaryotic ribosomes. Consequently, its role was underexplored in studies aiming at deciphering mechanisms involved in translational regulation. Several recent technical and conceptual breakthroughs have challenged this view and drawn attention to these mysterious modifications: first, the "specialized ribosome" concept, which has fueled new interest in ribosome functioning; and second, rRNA modification detection by omics-based mapping methods and by high-resolution structural techniques. Based on recent technical advances, new evidence supports this novel view of 2′-O-Me in different fields. Indeed, variability in 2′-O-Me and in the expression of 2′-O-methylation factors (FBL, C/D box snoRNAs) and in their contribution to particular cellular phenotypes have been investigated in various physiological contexts, including brain development [76,77] and stem cells [78], as well as pathological contexts, such as cancer [47,79,80] and genetic diseases [81]. 

In the context of translation, ribosomal diversity induced by 2′-O-Me heterogeneity stands to reason. Translation relies on a delicate molecular process that ensures not only the proper decoding of mRNA and peptide assembly but also the accommodation of a large variety of mRNAs. Consistently, it is not surprising that such a process relies on a variety of ribosomes. Recent publications on rRNA 2′-O-methylation [30], as well as rRNA pseudouridylation [73] and ribosomal proteins [82], have paved the way for further demonstrations of this novel concept.

Within chromatin, epigenetic regulatory mechanisms rely primarily on chemical modifications of both histones and DNA that modulate the use of the underlying DNA sequence. It is very tempting to establish a parallel with mRNA translation, and thus to propose that rRNA modifications represent a molecular support of an rRNA-mediated epitranscriptomic regulation of mRNA translation. The variation in 2′-O-Me profiles, the identification of potential hyper-sensitive 2′-O-Me sites [30,31], and the contribution of 2′-O-Me to the intrinsic ribosomal activities make 2′-O-Me an excellent candidate for rRNA epitranscriptomics. However, further studies should attempt to elucidate 2′-O-Me sites that promote—individually or in combination—translational regulation events of particular mRNAs. Adapting the composition of ribosome subpopulations would represent another means of regulating subfamilies of mRNAs, contributing to particular cellular pathways at the translational level.

Undoubtedly, recently developed tools to quantitatively analyze 2′-O-Me profiles, to visualize their role in the ribosome structure, and to evaluate translational regulation by ribosome profiling and in vitro translation will be instrumental in breaking the ribosome code.
